# *MTHFR* Gene Polymorphisms and DNA Methylation in Idiopathic Spontaneous Preterm Birth

**DOI:** 10.3390/medicina60122028

**Published:** 2024-12-09

**Authors:** Sanja Dević Pavlić, Roberta Šverko, Anita Barišić, Tea Mladenić, Jadranka Vraneković, Aleksandra Stanković, Ana Peterlin, Borut Peterlin, Saša Ostojić, Nina Pereza

**Affiliations:** 1 Department of Medical Biology and Genetics, Faculty of Medicine, University of Rijeka, 51000 Rijeka, Croatia; tea.mladenic@uniri.hr (T.M.); jadranka.vranekovic@uniri.hr (J.V.); sasa.ostojic@uniri.hr (S.O.); nina.pereza@uniri.hr (N.P.); 2 Department of Internal medicine, University Hospital Rijeka, 51000 Rijeka, Croatia; robertasverko@gmail.com; 3 Department of Gynecology and Obstetrics, University Hospital Rijeka, 51000 Rijeka, Croatia; anita.barisic@uniri.hr; 4 Department for Radiobiology and Molecular Genetics, Vinča Institute of Nuclear Sciences, National Institute of the Republic of Serbia, University of Belgrade, 11351 Belgrade, Serbia; alexas@vin.bg.ac.rs; 5 Institute of Histology and Embryology, Faculty of Medicine, University of Ljubljana, 1000 Ljubljana, Slovenia; ana.peterlin@mf.uni-lj.si; 6 Clinical Institute of Medical Genetics, University Medical Center Ljubljana, 1000 Ljubljana, Slovenia; borut.peterlin@kclj.si

**Keywords:** DNA methylation, genetic polymorphism, idiopathic spontaneous preterm birth, MTHFR, preterm birth

## Abstract

*Background and Objectives*: Preterm birth (PTB) is a complex condition with various contributing factors, including genetic and epigenetic influences such as DNA methylation. Methylenetetrahydrofolate reductase (MTHFR) plays a critical role in DNA methylation and the remethylation of homocysteine. This study aimed to investigate the association between maternal MTHFR C677T and A1298C polymorphisms, LINE-1 DNA methylation levels, and the risk of idiopathic spontaneous preterm birth (SPTB) in Caucasian women from Croatia and Slovenia. *Materials and Methods*: A total of 50 women with SPTB (<34 weeks of gestation) and 50 control women were included in the study. MTHFR polymorphisms were analyzed using polymerase chain reaction restriction fragment length polymorphism (PCR-RFLP), and LINE-1 DNA methylation levels were quantified using the MethyLight method. *Results*: The study found no significant differences in MTHFR C677T and A1298C polymorphisms’ genotype or allele frequencies between women with SPTB and controls. Additionally, no statistical significance of LINE-1 DNA methylation was found between the genotypes of the MTHFR polymorphisms analyzed. *Conclusions*: The study suggests no conclusive association between MTHFR C677T and A1298C polymorphisms, LINE-1 DNA methylation, and SPTB in Croatian and Slovenian women. Considering prior evidence connecting MTHFR polymorphisms, hyperhomocysteinemia, and PTB, the lack of homocysteine measurements and unassessed impact of folate or vitamin B supplementation limit the conclusions.

## 1. Introduction

Preterm birth (PTB) is defined as childbirth before 37 completed weeks of pregnancy or 259 days of gestation. It is the leading cause of perinatal morbidity and mortality, occurring in 5–18% of all births worldwide [1,2]. Preterm birth can be either medically induced due to pathological conditions related to the mother and/or the fetus, or spontaneous, which begins with contractions without rupture of the amniotic sac or premature rupture of the membranes at least one hour before beginning of labor [3]. In more than 50% of spontaneous preterm birth cases, the cause remains unknown and is considered a specific clinical syndrome called idiopathic spontaneous preterm birth (SPTB) [4]. There are multiple possible causes and pathological mechanisms involved in its pathogenesis, including inflammation and infection, uteroplacental ischemia and bleeding, and other local or general immune processes [5,6]. Interestingly, recent studies suggest the potential contribution of different genetic and epigenetic factors, such as DNA methylation [7,8,9].

Methylenetetrahydrofolate reductase (MTHFR) is one of the key enzymes in folic acid metabolism that is involved in methyl group synthesis and is important for remethylation of homocysteine into a methionine [10,11]. It is encoded by the MTHFR gene located on the short arm of chromosome 1. The two most extensively investigated functional polymorphisms in the MTHFR gene are: C677T and A1298C. The substitution of cytosine with thymine in position 677 of exon 4 or MTHFR C677T results in the substitution of the amino acid alanine with valine in position 226 in the protein, while the substitution of adenine with cytosine in the position 1298 of exon 7 or MTHFR A1298C results in the substitution of glutamate with alanine. Both variants result in a decrease of enzyme activity: MTHFR C677T increases the thermolability of the enzyme, reducing its activity by 50–70% in homozygotes and 30% in heterozygotes, while MTHFR A1298C reduces MTHFR enzyme activity to a lesser extent, but also more pronouncedly in homozygotes. Reduced enzyme activity leads to hyperhomocisteinemia, which can cause damage of blood vessels and increase the risk for PTB [10,11,12]. Previous research has shown that the most common MTHFR gene polymorphism affects DNA methylation either directly by interacting with folate status, or indirectly by affecting total homocysteine levels [13,14].

DNA methylation provides insight into genetic and environmental exposure, critical for many processes during lifetime and especially throughout embryonic development [15]. Although it is one of the most studied epigenetic mechanisms that coordinates gene expression, it is still unclear how it may affect PTB risk. Both global and site-specific DNA methylation patterns can be examined. One of the approaches which evaluates global DNA methylation through repetitive sequences includes long interspersed nucleotide elements 1 (LINE-1) DNA methylation, regarded as a surrogate of global DNA methylation. Moreover, there is evidence that genetic variants named methylation quantitative trait loci (meQTLs) could cause DNA hypomethylation or hypermethylation in various diseases [16]. Previously Barišić et al. showed no significant difference in LINE-1 DNA methylation between women with SPTB and the control group [17]. Moreover, they found no significant association between polymorphisms of DNA methyltransferase (*DNMT*) genes and LINE-1 DNA methylation. Since the contribution of MTHFR gene polymorphisms, which also represent meQTLs, and DNA methylation in PTB are poorly understood, our aim was to determine the impact of maternal MTHFR C677T and A1298C gene polymorphisms on LINE-1 DNA methylation and overall risk for SPTB in Croatian and Slovenian women.

## 2. Materials and Methods

### 2.1. The Subjects

This case-control study included 50 women who delivered spontaneously early preterm (23–33^6/7^ weeks of gestation) and 50 women in the control group who delivered at term. All included women were from Croatia and Slovenia and delivered during 2018 at Department of Obstetrics and Gynecology, University Medical Center in Ljubljana and at the Clinic of Obstetrics and Gynecology, Clinical Hospital Centre Rijeka, Croatia. Since the population of Croatia and Slovenia is predominantly Caucasian, all included participants belonged to white race. All women who met the criteria for inclusion in the study (either as subjects or controls) were offered the opportunity to participate in the research and, if they agreed, they consented by signing a written informed consent form at the time of delivery. After providing written informed consent, each participant completed a self-developed questionnaire filled out by an interviewer to collect demographic and clinical data. The study was conducted in accordance with the principles of the Declaration of Helsinki and has been approved by the Slovenian National Medical Ethics Committee (98/12/10, 2010) and the Ethics Committee for Biomedical Research of the Faculty of Medicine, University of Rijeka (2170-29-02/15-17-2, 2017). Demographic and clinical data of women with SPTB and their newborn children were collected in accordance with the data set for genetic epidemiology studies of PTB [7]. The number of participants was calculated according to the previously published literature on the frequencies of examined genotypes and alleles (the frequencies of minor alleles in the European, as well as in Croatian and Slovenian populations are 30% while the frequencies of homozygous genotypes are 10%) [18,19], using the online sample size and power calculator ClinCalc LLC (Chicago, IL, USA) with a study power of 80%.

All women with SPTB experienced spontaneous onset of preterm birth before 34 weeks of gestation in singleton pregnancies conceived naturally. Gestational age was determined based on the last menstrual period and verified through first-trimester ultrasound. If the gestational age estimates from the last menstrual period and ultrasound differed by more than seven days, the ultrasound measurement was used. None of the participants had known risk factors for preterm birth, such as diabetes, hypertension, kidney disease, autoimmune or allergic disorders, genital infections, in vitro fertilization, or other pregnancy complications. Neonates were free from congenital anomalies or signs of infection. Each SPTB case was matched with a control participant of similar age and parity who delivered a singleton baby at term following an uncomplicated pregnancy.

### 2.2. DNA Extraction and Genotype Analysis

Genomic DNA was extracted from peripheral blood leukocytes by standard procedure using a commercially available kit (Qiagen FlexiGeneDNA kit, Qiagen GmbH, Hilden, Germany) and stored at −20 °C. The MTHFR C677T and A1298C gene polymorphisms were genotyped using a polymerase chain reaction (PCR) based method combined with restriction fragment length polymorphism (RFLP) analysis. Primers, PCR, and RFLP conditions were modified according to the previously published literature [20].

For MTHFR C677T polymorphism, the expected size of restriction product for C allele was 198 base pairs (bp) and for T allele 175 bp and 23 bp. The polymorphism was analyzed using the following primers: MTHFR C677T—F (5′TGAAGGAGAAGGTGTCTGCGGGA3′) and MTHFR C677T—R (5′AGGACGGTGCGGTGAGAGTG3′). For the second MTHFR gene polymorphism, A1298C, the expected restriction product for A allele included five fragments of sizes 56 bp, 31 bp, 30 bp, 28 bp, and 18 bp and for C allele four fragments of sizes 84 bp, 31 bp, 30 bp, and 18 bp. This polymorphism was analyzed using the following primers: MTHFR A2198C—F (5′CTTT GGGGAGCTGAAGGACTACTAC3′) and MTHFR A2198C—R (5′CACTTTGTGACCATTCC GGTTTG3′).

Polymerase chain reaction (PCR) amplification was performed in a thermal cycler (Mastercycler personal, Eppendorf, Hamburg, Germany). PCR reactions were conducted in a total volume of 10 µL containing 10× PCR buffer, 25 mM MgCl, 10 mM dNTPs, 10x of each primer, 1 U Taq polymerase and 1 µL of template DNA. Amplification conditions included an initial denaturation step at 94 °C for 2 min, followed by 40 cycles of denaturation at 94 °C for 30 s, annealing at the 62 °C for 30 s, and extension at 72 °C for 30 s. A final extension step at 72 °C for 7 min was performed to ensure complete product formation.

The restriction digestion of PCR products was conducted using specific restriction enzymes selected to target regions of interest in the amplified fragments. Enzymes HinfI and MboII were used according to the manufacturer’s instructions for MTHFR C677T and A2198C, respectively. Each digestion reaction consisted of 10 µL of PCR product, 1× restriction enzyme buffer, 0.2 µL of the respective enzyme, and nuclease-free water to a final volume of 20 µL. Reactions were incubated at 37 °C for 2 h to ensure complete digestion. PCR products and restriction fragments were separated using electrophoresis on 3% agarose gels prepared with 1× TBE buffer. The gels were stained with 15 µL of GelRed (Olerup SSP, Saltsjöbaden, Sweden). A DNA ladder (1 kb or 100 bp, depending on product size; Thermo Fisher Scientific, Waltham, MA, USA) was included for size reference. Electrophoresis was performed at 80 V for approximately 1 h. The product bands were visualized under ultraviolet light.

### 2.3. Bisulfite Treatment and DNA Methylation Analysis

To distinguish between methylated and unmethylated cytosine residues bisulfite treatment was applied using EpiTectBisulfite Kit (Qiagen GmbH, Hilden, Germany). After bisulfite treatment, DNA was diluted 10X, resuspended in 30 µL of TE buffer, PCR-amplified and then sequenced to determine the methylation status of specific cytosines, thus revealing methylation patterns in the genome. Quantification of LINE-1 DNA methylation was done by applying the MethyLight methodology, designed and validated by Weisenberger et al. [21]. Method, primers, and procedure are described in detail in our previously published paper [17]. For data analysis, SDS 1.4.0 software (AppliedBiosystems, Forest City, CA, USA) was used. For the determination of LINE-1 DNAmethylation percentage, a modified formula for absolute quantification was applied, in a way that the final formula was as follows: (PMR/(PMR + PUR) × 100 [17,21].

### 2.4. Statistical Analysis

The statistical analysis was performed with Statistica for Windows, version 13.3 (StatSoft, Inc., Tulsa, OK, USA). The Hardy–Weinberg equilibrium was calculated using the Simple Hardy–Weinberg Calculator—Court Lab (Washington State University College of Veterinary Medicine, Pullman, WA, USA).

Differences in genotype and allele frequencies of the MTHFR C677T and A2198C polymorphism between women with SPTB and controls were calculated using the Pearson chi-square test (χ^2^). The association between the investigated polymorphisms and susceptibility to SPTB was assessed using dominant, recessive, and codominant genetic models using odds ratio (OR) and 95% CI.

The distribution of numerical variables was assessed using the Kolmogorov–Smirnov test for normality. For normally distributed data, the median was used for numerical variables, and for data with deviations from the normal distribution the arithmetic mean was used as well as nonparametric methods. One-way analysis of variance (ANOVA) was used to compare the mean maternal age at delivery between the different genotypes, while the Kruskal–Wallis test was used to compare the mean fetal birth weight between the genotypes. The differences between the rest of maternal/newborn characteristic were calculated using χ^2^ test. Differences in LINE-1 DNA methylation levels across different MTHFR genotypes within the SPTB and control groups were tested using the Kruskal–Wallis test.

Statistical significance was set at a *p*-value of less than 0.05.

## 3. Results

Maternal and newborn characteristics are shown in Table 1.

The genotype distribution of both MTHFR C677T and A1298C polymorphisms in women with SPTB and controls conformed to Hardy–Weinberg equilibrium (*p* < 0.050). Table 2 presents the genotype and allele frequencies for these polymorphisms in both groups, revealing no significant differences between women with SPTB and the control group.

Similarly, no statistically significant differences were observed across different inheritance models (dominant, recessive, or codominant) between the two groups (Table 3).

Furthermore, no significant correlations were found between MTHFR genotypes and clinical characteristics, including maternal age at delivery, gestational age, birth weight, smoking status before and during pregnancy, or prior/familial history of PTB, among women with SPTB (Table 4).

**Figure 1:**
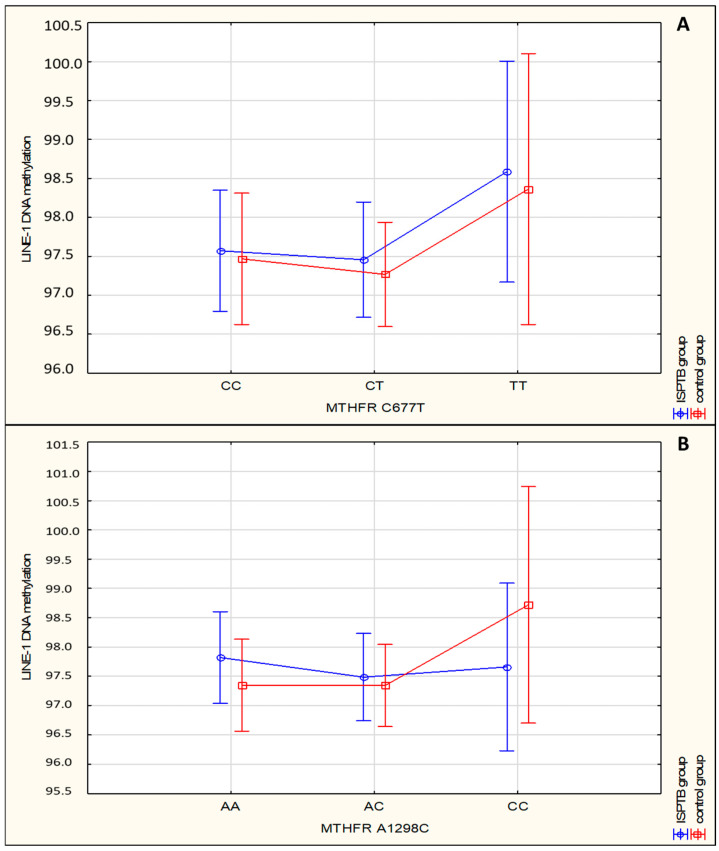
Association of LINE-1 DNA methylation and MTHFR C677T (**A**) and MTHFR A2198C (**B**) polymorphism genotypes in women with SPTB and in the control group (*p* > 0.050).

Moreover, statistical analysis showed no significant difference in LINE-1 DNA methylation between different genotypes of both investigated MTHFR polymorphisms (Table 5, Figure 1).

## 4. Discussion

This case-control study was conducted to determine the possible association between maternal MTHFR C677T and A1298C gene polymorphisms with LINE-1 DNA methylation and SPTB in Croatian and Slovenian women. We found no significant difference in the distribution of both MTHFR polymorphisms’ genotypes and alleles between women with SPTB and the control group. Furthermore, the investigated polymorphisms were not associated with examined clinical characteristics of the subjects. Additionally, there was no significant association between MTHFR C677T and A2198C genotypes and DNA methylation (Figure 1).

Several studies and meta-analyses have previously analyzed the correlation between MTHFR polymorphisms and predisposition to PTB, yielding inconsistent results [11,12,22,23]. Most suggest that elevated homocysteine levels, particularly due to MTHFR C677T, may be the cause. Hyperhomocysteinemia primarily affects blood vessels, damaging placental endothelium and increasing pregnancy complication risk through induction of oxidative stress, arteriolar constriction, and disruption of the placental coagulant/anticoagulant balance [24,25].

Most studies that investigated the association between MTHFR C677T and PTB with positive and significant results included Asian women. One of the most cited case-control studies was conducted on 216 women from China, with 108 of them having positive history of PTB [22]. Their results reached statistical significance with higher occurrence of TT genotype as well as T allele for MTHFR C677T in women with PTB than in the control group (*p* = 0.004 and *p* = 0.002, respectively). Similar studies in India showed statistically significant higher distribution of MTHFR C677T polymorphism in women with PTB [12]. Contrary to the above, a study from South Korea examined the correlation of both MTHFR C677T and A1298C polymorphisms with PTB and showed that both polymorphisms could have protective effects for PTB in Korean women [23].

A meta-analysis by Wu et al. included women of different ethnicities with a positive history of PTB and consisted of 25 separate studies, 8 of which examined the association between maternal MTHFR C677T and PTB and low gestational weight [11]. Detailed analysis of their results according to ethnicity showed that the most positive correlation between TT homozygote genotype and PTB was found in Asian women. Importantly, all studies conducted have shown different prevalence of MTHFR polymorphisms depending on ethnicity and geographical location. This could be the reason why our research, conducted on the European Caucasian population (Croatian and Slovenian women) did not show similar results.

Homocysteine levels are influenced by epigenetic regulation as well. Precisely, hyperhomocisteinemia correlates with the lower activity of DNA methylation in specific places in genome 14, which can increase the risk of PTB. Hypomethylation itself might contribute to PTB through the process of inflammation by triggering the innate immunity pathway or by affecting the cellular function through stimulation of transcription of sequences activated during conditions of cellular stress [26]. Up until now, only three studies have evaluated methylation in peripheral blood leukocytes of mothers with PTB [8,27,28]. Burris et al. [28] demonstrated that higher LINE-1 DNA methylation in maternal blood at early pregnancy period assumes reduced risk of PTB, while Hong et al. [8] and Parets et al. [27] identified differentially methylated maternal CpG sites that may serve as biomarkers for early PTB and various adulthood diseases of respective fetus.

Homocysteine levels are affected not only by MTHFR C677T and A1298C genotype or DNA methylation but also by folic acid and B vitamin intake. Taking folic acid and/or B vitamin into account may explain why different studies have come to different conclusions when trying to prove a link between methylation, MTHFR polymorphisms and SPTB. Since most people today do not consume the required dose of vitamin B in their diet, supplementation with folic acid and B-complex vitamins (B12, B6 and B2) could help normalize homocysteine levels and mitigate the effects of MTHFR polymorphisms on PTB [29,30]. A recent study by Bošković et al. emphasized that although MTHFR gene mutations were highly prevalent in pregnant women, their influence on homocysteine levels in this population could be mitigated by vitamin supplementation [31]. This emphasizes the importance of an adequate intake of folic acid and vitamin B12 during pregnancy to regulate homocysteine levels [32].

One of the primary limitations of this study is the relatively small sample size, which may have reduced the ability to detect significant associations between MTHFR polymorphisms and SPTB. A larger cohort would provide more robust data and enhance the generalizability of the findings. Future studies should include a larger sample size to confirm these results and improve the reliability of the conclusions. Additionally, exploring other genes involved in the methylation process through both targeted and untargeted approaches would offer a more comprehensive understanding of genetic factors contributing to SPTB. The assessment of plasma levels of folic acid and homocysteine would provide valuable insights into the role of these biomarkers in SPTB risk. Furthermore, validated questionnaires assessing dietary folic acid intake and the use of folic acid supplements would contribute to a more comprehensive analysis of the impact of nutrition on pregnancy outcomes.

## 5. Conclusions

Although our results suggest no significant association between MTHFR C677T and A1298C polymorphisms, LINE-1 DNA methylation, and SPTB in Croatian and Slovenian women; the precise relationship between MTHFR polymorphisms, DNA methylation, and SPTB remains inconclusive. Further studies are needed to fully understand their roles, and future research should encompass a broader range of genetic and epigenetic factors to provide a more comprehensive understanding of SPTB.

## Figures and Tables

**Table 1:** Maternal and newborn characteristics.

Maternal Characteristics	SPTB Group	Control Group	*p*
Maternal age (years)	Median (range)	Median (range)	
	30 (17–42)	31 (20–42)	0.375
Gestational age at delivery (weeks)	Mean ± SD	Mean ± SD	
	30.7 ± 3.0	39.5 ± 1.0	<0.001 *
Smoking before pregnancy	N (%)	N (%)	
Yes	16 (32%)	9 (20%)	0.185
No	34 (68%)	36 (80%)	
Smoking during pregnancy	N (%)	N (%)	
Yes	7 (14%)	5 (11%)	0.672
No	43 (86%)	40 (89%)	
Previous PTB	N (%)	N (%)	
Yes	5 (10%)	1 (2%)	0.119
No	45 (90%)	44 (98%)	
Familial PTB	N (%)	N (%)	
Yes	16 (32%)	1 (2%)	<0.001 **
No	34 (68%)	44 (98%)	
Newborn characteristics	Patients	Control subjects	*p*
Newborn weight (g)	Mean ± SD	Mean ± SD	
	1764.1 ± 556.1	3475.1 ± 340.6	<0.001 ***

* SE 0.45; 95% CI 7.91–9.69; ** OR 20.71; 95% CI 2.61–163.98; *** SE 92.22; 95% CI 1527.99–1894.01.

**Table 2:** MTHFR gene polymorphisms’ genotype and allele frequencies in the SPTB (N = 50) and the control group (N = 50).

		SPTB Group/N (%)	Control Group/N (%)	χ^2^	*p*
MTHFR C677T					
Genotype	CC	21 (42.0)	18 (36.0)		
	CT	23 (46.0)	27 (54.0)		
	TT	6 (12.0)	5 (10.0)	0.64	0.726
Allele	C	65 (65.0)	63 (63.0)		
	T	35 (35.0)	37 (37.0)	0.09	0.768
MTHFR A1298C					
Genotype	AA	21 (42.0)	22 (44.0)		
	AC	23 (46.0)	25 (50.0)		
	CC	6 (12.0)	3 (6.0)	1.11	0.575
Allele	A	65 (65.0)	69 (69.0)		
	C	35 (35.0)	31 (31.0)	0.36	0.547

**Table 3:** MTHFR gene polymorphisms under different genetic models in ISPTB (N = 50) and control groups (N = 50).

Genetic Models	ISPTB Group	Control Group	OR	95% CI	*p*
MTHFR C677T					
CT+TT/CC	29/21	32/18	0.78	0.35–1.74	0.539
TT/CT+CC	6/44	5/45	1.28	0.35–4.32	0.749
TT/CC	6/21	5/18	1.03	0.27–3.94	0.967
CT/CC	23/21	27/18	0.73	0.31–1.69	0.463
MTHFR A1298C					
AC+CC/AA	29/21	28/22	1.09	0.49–2.40	0.840
CC/AC+AA	6/44	3/47	2.14	0.50–9.07	0.303
CC/AA	6/21	3/22	2.10	0.46–9.48	0.337
AC/AA	23/21	25/22	0.96	0.42–2.20	0.930

**Table 4:** Associations between MTHFR C677T and A1298C genotypes and clinical characteristics of SPTB subjects and their newborns.

Maternal/Newborn Characteristics	MTHFR C677T		MTHFR A1298C	
	χ^2^	*p*	χ^2^	*p*
Gestational age at delivery	5.15	0.272	4.42	0.352
Smoking before pregnancy	3.02	0.221	1.30	0.522
Smoking during pregnancy	2.47	0.291	2.47	0.291
Previous PTB	0.91	0.635	0.35	0.842
Familial PTB	0.05	0.976	1.28	0.527
	t	*p*	t	*p*
Maternal age	−0.17	0.863	−1.77	0.089
Newborn weight	−1.18	0.246	−0.28	0.783

**Table 5:** Differences in LINE-1 DNA methylation between genotypes of MTHFR C677T and A1298C polymorphisms in the SPTB and control groups.

LINE-1 DNA Methylation		SPTB Group/Mean (SD)	Control Group/Mean (SD)	χ^2^	*p*
MTHFR C677T					
	CC	97.571 (1.210)	97.468 (2.055)		
	CT	97.456 (1.712)	97.267 (2.035)		
	TT	98.588 (0.821)	98.364 (1.635)	2.14	0.344
MTHFR A1298C					
Genotype	AA	97.819 (1.373)	97.347 (1.789)		
	AC	97.484 (1.591)	97.341 (2.177)		
	CC	97.659 (1.329)	98.719 (1.944)	0.40	0.818

## Data Availability

The datasets generated and analyzed as part of the current study are not publicly accessible, as the patients in this study have given informed consent, which does not allow the data to be published in public databases. However, the data are available upon reasonable request. Requests should be addressed to corresponding author.
